# Fully automated volumetric modulated arc therapy technique for radiation therapy of locally advanced breast cancer

**DOI:** 10.1186/s13014-023-02364-8

**Published:** 2023-10-30

**Authors:** Livia Marrazzo, Laura Redapi, Roberto Pellegrini, Peter Voet, Icro Meattini, Chiara Arilli, Silvia Calusi, Marta Casati, Deborah Chilà, Antonella Compagnucci, Cinzia Talamonti, Margherita Zani, Lorenzo Livi, Stefania Pallotta

**Affiliations:** 1 Department of Experimental and Clinical Biomedical Sciences “Mario Serio”, University of Florence, Florence, Italy; 2 Medical Physics Unit, Azienda Ospedaliero-Universitaria Careggi, Florence, Italy; 3 Medical Affairs & Research Clinical Liaison, Elekta AB, Stockholm, Sweden; 4 Radiation Oncology Unit, Oncology Department, Azienda Ospedaliero-Universitaria Careggi, Florence, Italy; 5 Medical Physics Unit, Azienda USL Toscana Centro, Pistoia-Prato, Italy

**Keywords:** Radiotherapy, Automated planning, VMAT, Locally advanced Breast cancer, Multicriterial optimization

## Abstract

**Background:**

This study aimed to evaluate an a-priori multicriteria plan optimization algorithm (mCycle) for locally advanced breast cancer radiation therapy (RT) by comparing automatically generated VMAT (Volumetric Modulated Arc Therapy) plans (AP-VMAT) with manual clinical Helical Tomotherapy (HT) plans.

**Methods:**

The study included 25 patients who received postoperative RT using HT. The patient cohort had diverse target selections, including both left and right breast/chest wall (CW) and III-IV node, with or without internal mammary node (IMN) and Simultaneous Integrated Boost (SIB). The Planning Target Volume (PTV) was obtained by applying a 5 mm isotropic expansion to the CTV (Clinical Target Volume), with a 5 mm clip from the skin. Comparisons of dosimetric parameters and delivery/planning times were conducted. Dosimetric verification of the AP-VMAT plans was performed.

**Results:**

The study showed statistically significant improvements in AP-VMAT plans compared to HT for OARs (Organs At Risk) mean dose, except for the heart and ipsilateral lung. No significant differences in V_95%_ were observed for PTV breast/CW and PTV III-IV, while increased coverage (higher V_95%_) was seen for PTV IMN in AP-VMAT plans. HT plans exhibited smaller values of PTV V_105%_ for breast/CW and III-IV, with no differences in PTV IMN and boost. HT had an average (± standard deviation) delivery time of (17 ± 8) minutes, while AP-VMAT took (3 ± 1) minutes. The average γ passing rate for AP-VMAT plans was 97%±1%. Planning times reduced from an average of 6 h for HT to about 2 min for AP-VMAT.

**Conclusions:**

Comparing AP-VMAT plans with clinical HT plans showed similar or improved quality. The implementation of mCycle demonstrated successful automation of the planning process for VMAT treatment of locally advanced breast cancer, significantly reducing workload.

**Supplementary Information:**

The online version contains supplementary material available at 10.1186/s13014-023-02364-8.

## Background

In recent years, automatic planning (AP) has been introduced to reduce planning workload, minimize inter-operator variability, and enhance the quality of plans. A comprehensive review by Hussein et al. [1], effectively summarizes the available solutions, identifying three main categories of AP approaches: knowledge-based planning, protocol-based automatic iterative optimization, and multicriteria optimization (MCO) driven by pareto-navigation (a-posteriori) or automated (a-priori).

Breast cancer is the most common tumor among women, accounting for 13.3% of all new cancer cases diagnosed in European Union Countries in 2020 [2]. Radiotherapy (RT) is an important treatment component for breast cancer patients, following either conservative or radical surgery [3–5]. Implementing planning automation in breast cancer treatment is thus expected to have a large impact on workload, quality, and standardization [6, 7].

Target volumes in breast radiotherapy vary based on factors like stage, tumor biology, risk factors, nodal involvement, and surgery extent [8]. Typically, they cover the entire breast or chest wall and may extend to nodal regions. Tangential fields are standard for whole breast treatment, while more complex rotational techniques like VMAT or Helical Tomotherapy (HT) are used when nodal involvement occurs [9]. These techniques eventually allow for a simultaneous integrated boost (SIB) due to their dose modulation capability [10]. Manual treatment planning for these advanced techniques and complex targets can be a very challenging and time-consuming process, which pushed the development of AP techniques for this type of treatment [11, 12].

In this study, we investigated the performance of an a priori-MCO plan optimization algorithm implemented in the research version of a commercial TPS, for the treatment of locally advanced breast cancer patients, by comparing the fully automatically generated VMAT (Volumetric Modulated Arc Therapy) plans with previously generated manual clinical HT plans. VMAT has the advantages of shorter delivery times when compared to HT. The goal is to develop a single robust configuration of the algorithm that can be successfully applied to all patients, regardless of their anatomical variability, treatment side, and target heterogeneity.

## Methods

### Patients

Twenty-five consecutive patients who received postoperative RT using HT were included in the study (validation set). Most (23 out of 25) had a prescription dose of 50 Gy in 25 fractions, while 2 were treated with a SIB (57 Gy in 25 fractions).

The median age of the series at the time of breast cancer diagnosis was 49.8 years (mean 49.6; range 36–68). The majority of patients had pT1-2 (56%), pN2-3 (60%), hormonal receptor-positive (72%), and HER2-negative (72%) breast cancer. Most patients received neo(adjuvant) chemotherapy (92%) and adjuvant endocrine therapy (72%). The main clinical characteristics are summarized in Table S1.

Patients were positioned supine with their arms above their heads using either a WingSTEP™ (Elekta AB, Stockholm, Sweden) or a Wing Board (CIVCO Inc., Coralville, IA) and underwent scanning with a Philips Big Bore CT (3 mm slice thickness).

Clinical Target Volumes (CTV) were delineated according to the ESTRO contouring guidelines for the breast, chest wall (CW), III-IV axillary nodes (III-IV), and IMNs [13]. The Organs At Risk (OARs) included the heart, lungs, spinal cord, esophagus, and contralateral breast. The Planning Target Volume (PTV) was obtained by applying a 5 mm isotropic expansion to the CTV, with a 5 mm clip from the skin to account for the lack of electronic equilibrium.

The patient cohort exhibited heterogeneity in target selection, including 9 left and 5 right CW plus III-IV nodes, 2 left and 7 right CW and III-IV plus IMNs irradiation, and 1 left and 1 right breast and III-IV with SIB (57 Gy in 25 fractions). The mean PTV volumes were: 656 ± 305 cm^3^ (236–1336) for PTV breast/CW, 91 ± 35 cm^3^ (23–155) for PTV III-IV, 85 ± 107 cm^3^ (32–364) for PTV IMN and 125 cm^3^ for PTV boost.

### Tomotherapy planning

Manual HT treatment plans were generated using TomoHD™ TPS (V5.1.1.6, Accuray®, USA). HT plans were created with a field size of 2.5 cm, while the pitch was selected based on the work of Chen et al. [14], and varied between patients, as did the modulation factor. The pitch values varied in the range of 0.264–0.436, while the actual modulation factor varied in the range of 1.610–3.501 (with an average value of 2.433). No dynamic jaws option was used since it is not available in our HT machine. A cylindrical help structure placed medially was used and completely blocked to minimize dose to OARs and direct beamlets tangentially through the PTV of the breast or CW.

### m-Cycle

AP-VMAT plans were obtained with the novel system for fully automated multi-criterial generation of deliverable VMAT plans implemented in Monaco TPS and based on lexicographic multi-criterial optimization named mCycle. The algorithm generates a single Pareto-optimal plan per patient, and the system must be configured a priori for each treatment protocol to ensure that the generated plans are clinically favorable. This configuration is achieved by defining a lexicographic “wish-list” with predefined clinical and planning constraints and prioritized objectives for OARs and PTVs based on the clinical protocol.

The a priori-MCO approach was initially implemented at the Erasmus MC Cancer Center Institute in software called iCycle [15]. In its initial implementation, the software generated an optimized fluence map, which needed to be “translated” into a Monaco template to obtain a segmented deliverable plan. The iCycle system has been evaluated for several treatment sites, including prostate [16, 17], cervical cancer [18], lung [19], head and neck [20], and low-risk breast cancer [21], all of which demonstrated the overall superiority of a priori-MCO compared to manual planning.

The implementation of the iCycle algorithm into Elekta Monaco involved providing the optimization algorithm with the same cost functions as Monaco and utilizing the same Monte Carlo dose calculation algorithm [22].

In the wish-list any term of the cost function is specified with a priority which can be a Clinical Constraint, a Planning Constraint, or an Objective Priority, with #1 indicating the highest priority, typically assigned to target coverage. In mathematical terms, these priorities are associated with the weights of the cost functions used during the optimization process. Clinical Constraints receive the highest weight, as violations of these prescriptions would result in the plan’s non-acceptance. Planning Constraints represent prescriptions that planners typically follow in terms of gradient requirements, while Objective Priorities are prescriptions that, when satisfied, are converted into constraints, and are not allowed to be violated during the optimization process.

Five guidelines have been followed for the creation of the robust with-list based on the Institute clinical protocol:

1. A protocol item, whose violations lead to plan rejection is a hard Clinical Constraint.
2. All additional dose gradient prescriptions (global conformality) are asserted as Planning Constraints.
3. All target prescriptions should be accounted for as 1st priority objective.
4. All other protocol prescriptions are translated into lower priority objectives.
5. Further secondary objectives can be declared as lower order priorities.

To develop the AP-VMAT wish-list, the PTVs and OARs ideal dosimetric criteria of the internal clinical protocol were used as starting point (Table 1). For reproducing the same quality of HT clinical plans, protocol constraints were modified using the statistics of 5 further locally advanced breast cancer patients (training set) treated with HT (all left sided, with no SIB and no IMN), which is reported in the second column of Table 1. As can be observed, deviations from the protocol’s ideal constraints are sometimes accepted, given that the cases treated with HT often involve particularly challenging anatomies. While the clinical protocol was used to assign the priority levels in the wish-list, the OARs dose parameters of the training set were adopted at the upper limit of their standard deviations to inspire the values allowing the most robust possible description of the wish-list and leaving to MCO to make them patient specific during the optimization.

The process of wish-list creation is based on an iterative process, where, for each change, the wish-list is retested on the training dataset. The advantage of such a procedure is that it doesn’t require a big amount of carefully selected data, whereas the disadvantage is that the iterative process might take few days to be completed over the training dataset.

All AP-VMAT plans were generated using a 6MV photon beam from an Elekta VersaHD linac equipped with an Agility multileaf collimator.

**Table 1:** PTVs and OARs dosimetric criteria of the internal protocol (ideal values) and PTVs and OARs statistics of the same parameters extracted from the training set

Structure	Internal protocol ideal dosimetric criteria	Dosimetric Average Values (±1sd and range) in the training set
PTV boost	V54.15 Gy > 95%	
	V58.85 Gy < 1%	
PTV breast/CW	V52.5 Gy < 10%	V52.5 Gy = 0.7±1.2 (0.0-2.5) (%)
	V47.5 Gy > 95%	V47.5 Gy = 96±1 (95–97) (%)
	Dmax < 55 Gy	D1%= 52±1 (51–53) (Gy)
PTV III e IV	V52.5 Gy < 10%	V52.5 Gy = 0.1±0.2 (0.0-0.4) (%)
	V47.5 Gy > 95%	V47.5 Gy = 96±1 (94–97) (%)
	Dmax < 55 Gy	D1%= 51.7±0.3 (51.5–52.0) (Gy)
PTV IMN	V52.5 Gy < 10%	
	V47.5 Gy > 95%	
	Dmax < 55 Gy	
Heart	Dmean < 3 Gy	Dmean = 3.9±0.8 (3.2–4.8) (Gy)
Contralateral breast	Dmean < 7 Gy	Dmean = 7.8±1.5 (6.8–10.1) (Gy)
	D0.03 cm³<26 Gy	D0.03 cm³= 27±10 (15–39) (Gy)
Ipsilateral lung	V30Gy < 10%	V30Gy = 10±2 (10–13) (%)
	V20Gy < 20%	V20Gy = 20±4 (17–26) (%)
	V5Gy < 50%	V5Gy = 45±11 (34–55) (%)
Contralateral lung	V5Gy < 10%	V5Gy = 22±16 (3–42) (%)
	Dmean < 3 Gy	Dmean = 3±2 (1–5) (Gy)
Esophagus	Dmean < 35 Gy	Dmean = 8±2 (6–11) (Gy)
	V45Gy < 33%	V45Gy = 1.4±2.5 (0–5) (%)
Cord		Dmean = 3±1 (2–4) (Gy)
	Dmax < 45 Gy	Dmax = 23±9 (10–29) (Gy)

### Deliverability of the automatically generated plans

To verify the deliverability of the automatically generated plans, QA measurements were performed using ArcCHECK® (Sun Nuclear Corporation, Melbourne, FL). The agreement between measured and calculated dose distributions was evaluated in terms of γ passing rate (3%, 2 mm, global).

### Plan comparison and evaluation

A comparison of selected dosimetric parameters between HT and AP-VMAT plans was conducted. DICOM data (CT, RTStructure, RTPlan, and RTDose) were exported to ProKnow DS (version 1.33.0, Elekta AB, Stockholm, Sweden), which was used for extracting the dosimetric parameters.

Paired two-sided Wilcoxon’s signed-rank tests (significance level 0.05) were employed to assess statistical significance. Statistical analysis was performed using OriginPro (version 9.0.0, OriginLab Corporation, Northampton, MA).

## Results

It was possible to generate a single wish-list for all patients (Table S2) with only minor modifications required to incorporate the differences in targets and doses and, in particular, to account for the presence of a SIB (Table S3). Plan parameters used in AP-VMAT plans (calculation properties, fluence optimization, shape optimization and sequencing) are reported in Table S4.

**Fig. 1:**
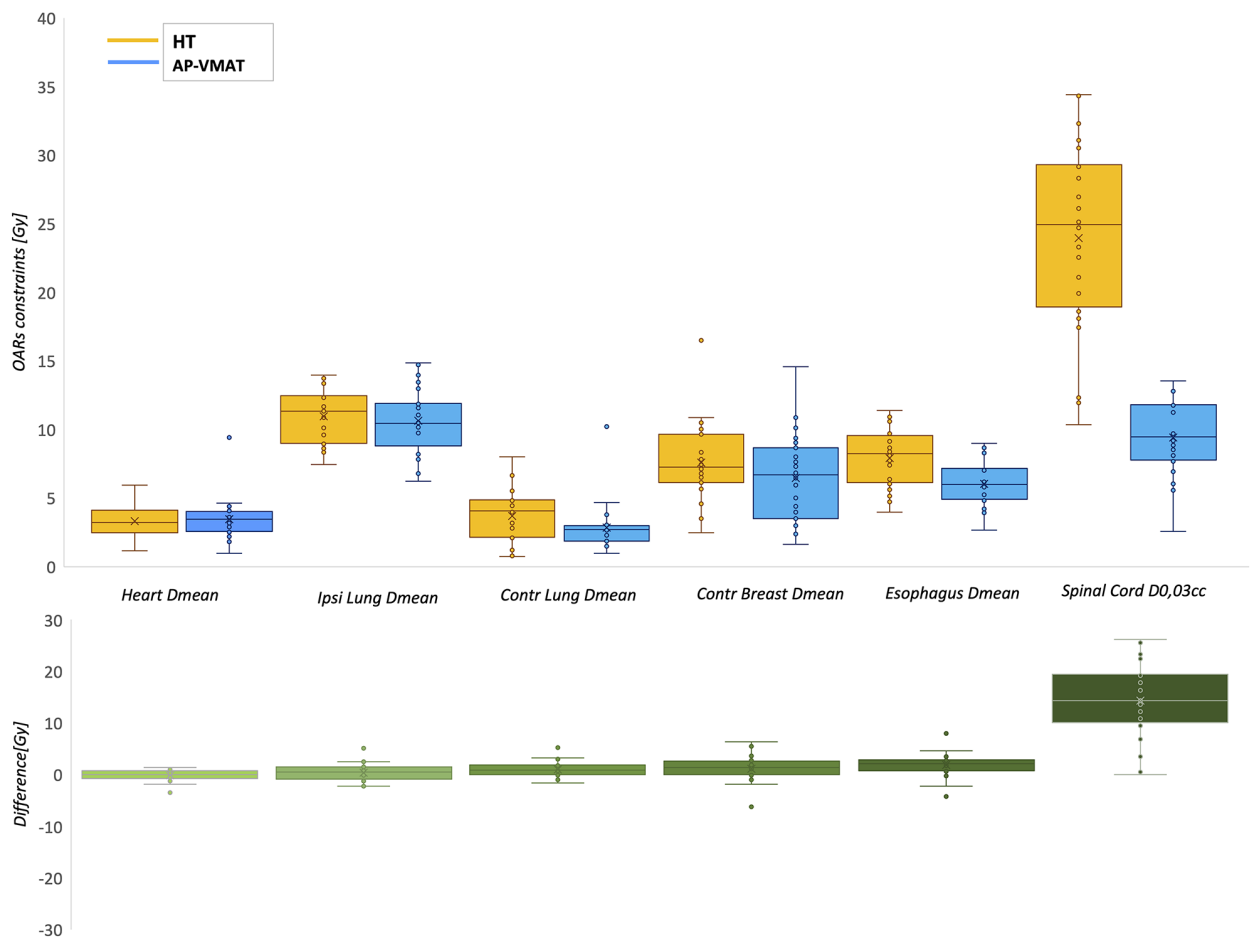
Mean OARs doses and spinal cord D_0.03 cc_ for the two techniques (HT in yellow, AP-VMAT in blue). The boxplot of the differences is reported in green

**Fig. 2:**
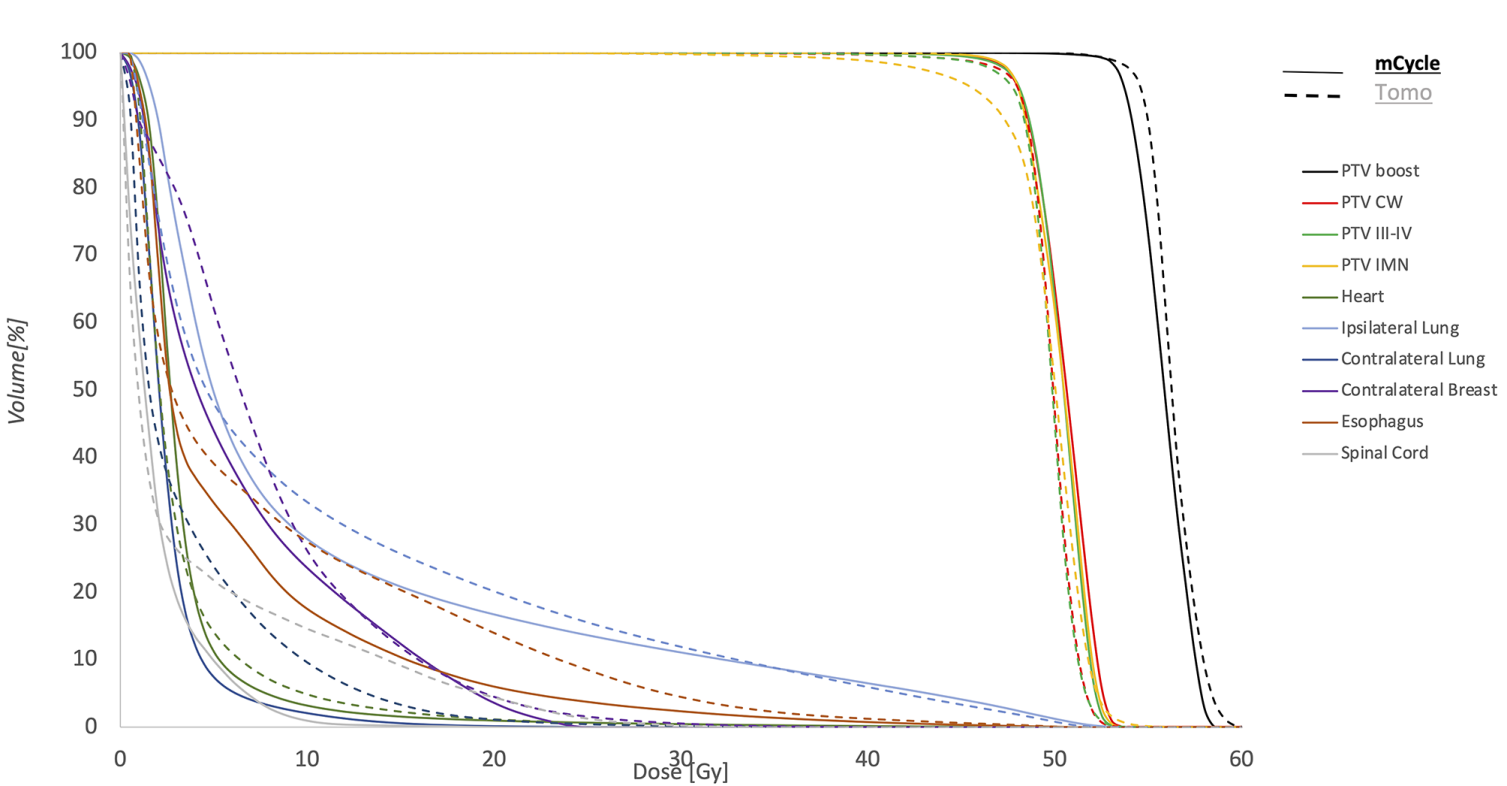
Population mean DVHs for the two techniques (HT dashed and AP-VMAT solid)

**Fig. 3:**
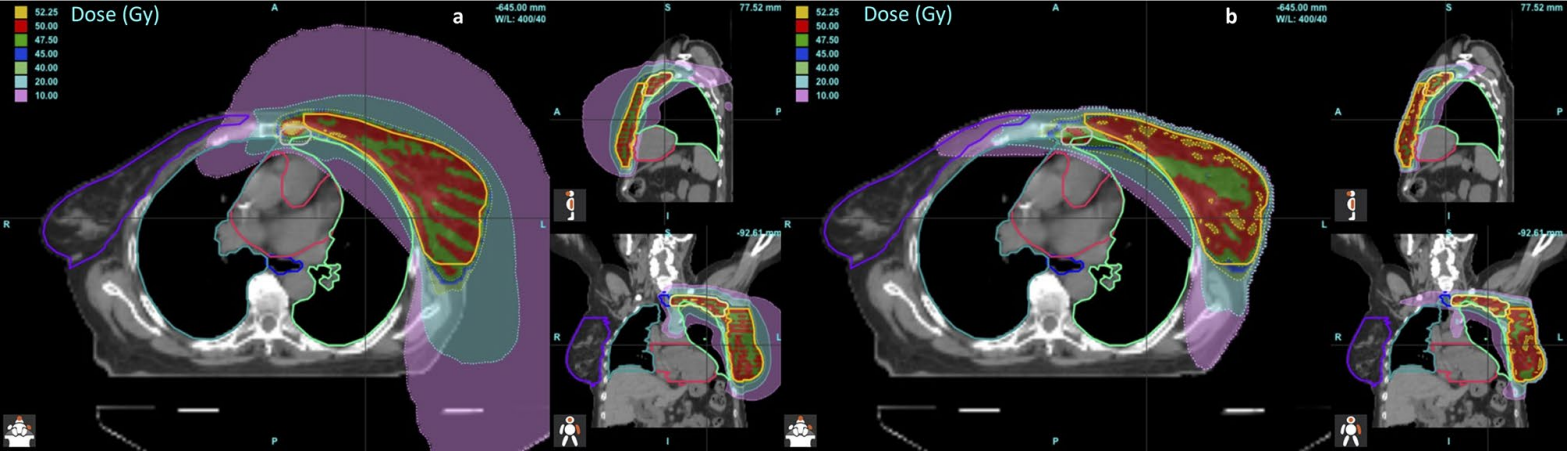
Dose distribution for a representative patient: (**a**) HT and (**b**) AP-VMAT plans

**Fig. 4:**
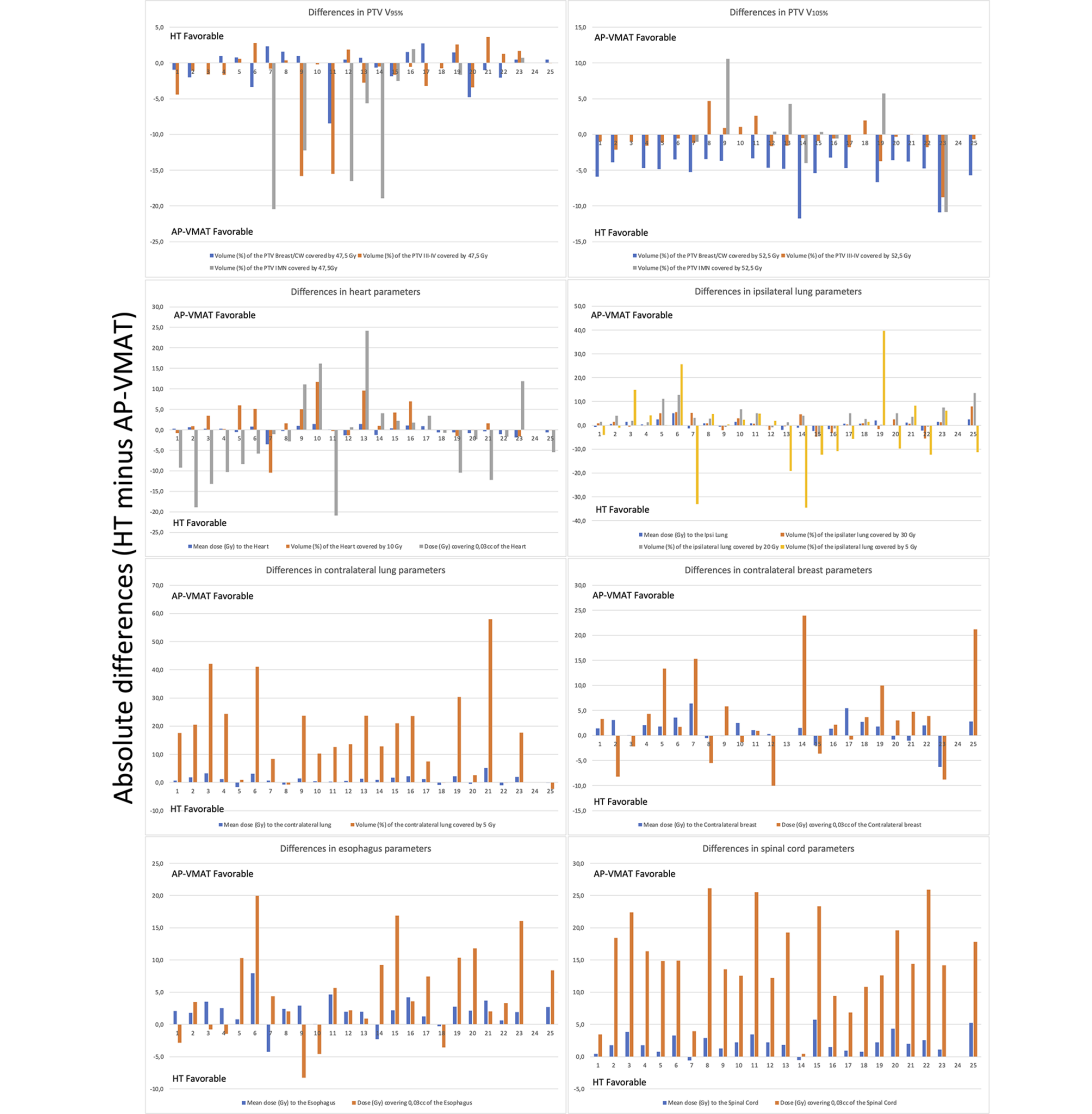
For all 25 patients, differences between HT and AP-VMAT (expressed as HT minus AP-VMAT) with positive values representing better quality for AP-VMAT (except for PTV V95%)

Statistically significant improvements were observed for AP-VMAT compared to HT in terms of OARs D_mean_ (p < 0.05), except for the heart and ipsilateral lung (Fig. 1). Regarding the PTVs, there were no statistically significant differences in V95% for PTV breast/CW and PTV III-IV, while an increase in coverage for PTV IMN was observed (p < 0.05) in AP-VMAT plans compared to HT (Table 2; Fig. 2). As for PTV V_105%_, HT plans exhibited smaller values for PTV breast/CW and PTV III-IV (averaging 4% ± 3% and 1% ± 2%, respectively), while no differences were observed for PTV IMN and PTV boost, as can be observed by examining the dose distribution (Fig. 3). Details on the comparisons between HT and AP-VMAT plans, for each of the 25 patients of the validation set, are presented in Fig. 4.

The delivery times of HT plans exhibited wide variation, with an average of 1000.6 s (570.1–1634.3).

Overall, AP-VMAT delivery times were less variable, with an average of 192 s (162–222). The average γ passing rate for the AP-VMAT plans was 97%±1%. Planning times were reduced from an average of 6 h for HT to approximately 1 h for AP-VMAT (around 2 min to prepare the plan and launch the calculation and 1 h of calculation time, which does not require any user involvement).

**Table 2:** Plan parameters for manually generated Tomotherapy plans (HT) and automatically generated VMAT plans (AP-VMAT).

	parameter		mean value ± 1 SD	range	p-value
PTV breast/CW	V_95%_ [%]	HT	97 ± 2	90–100	0.7
		AP-VMAT	97 ± 1	95–100	
	D_1%_ [Gy]	HT	**52.1 ± 0.6**	51.1–53.2	**0.01**
		AP-VMAT	52.9 ± 0.4	51.5–53.4	
	V_105%_ [%]	HT	**0.7 ± 0.8**	0.02–2.48	**0.003**
		AP-VMAT	5.6 ± 2.7	0.1–13.2	
PTV III-IV	V_95%_ [%]	HT	97 ± 2	79–100	0.2
		AP-VMAT	97 ± 2	93–100	
	V_105%_ [%]	HT	**1.0 ± 1.5**	0.0-5.6	**0.02**
		AP-VMAT	1.7 ± 1.8	0.0-8.8	
PTV IMN	V_95%_ [%]	HT	90 ± 8	78–99	**< 0.001**
		AP-VMAT	**98 ± 2**	95–100	
	V_105%_ [%]	HT	3.4 ± 3.6	0.7–11.3	0.3
		AP-VMAT	2.9 ± 4.1	0.3–13.0	
PTV boost	V_95%_ [%]	HT	97 ± 2	96–98	
		AP-VMAT	95 ± 3	93–97	
	V_105%_ [%]	HT	2.0 ± 2.8	0.0–4.0	
		AP-VMAT	0.46 ± 0.01	0.45–0.47	
Heart	D_mean_ [Gy]	HT	3.2 ± 1.3	1.2–5.9	0.7
		AP-VMAT	3.4 ± 1.5	1.0-9.4	
	V_10Gy_ [%]	HT	4.9 ± 4.5	0-14.3	0.06
		AP-VMAT	3 ± 5	0–25	
	D_0.03 cc_ [Gy]	HT	28 ± 15	3–52	0.6
		AP-VMAT	30 ± 17	4–53	
Ipsilateral lung	D_mean_ [Gy]	HT	11 ± 2	7–14	0.3
		AP-VMAT	11 ± 2	6–15	
	V_5Gy_ [%]	HT	49 ± 11	34–91	0.7
		AP-VMAT	50 ± 13	29–81	
	V_20Gy_ [%]	HT	20 ± 5	10–29	0.09
		AP-VMAT	17 ± 5	7–25	
	V_30Gy_ [%]	HT	12 ± 4	5–22	0.5
		AP-VMAT	11 ± 4	4–18	
Contralateral lung	D_mean_ [Gy]	HT	3.6 ± 1.9	0.7-8.0	**0.004**
		AP-VMAT	**2.6 ± 1.0**	1.0-4.8	
	V_5Gy_ [%]	HT	24 ± 17	0–65	0.3
		AP-VMAT	8 ± 8	0–33	
Contralateral breast	D_mean_ [Gy]	HT	8 ± 3	3–17	**0.01**
		AP-VMAT	**6 ± 3**	2–15	
	D_0.03 cc_ [Gy]	HT	28 ± 9	14–50	0.3
		AP-VMAT	24 ± 2	18–26	
Esophagus	D_mean_ [Gy]	HT	8 ± 2	4–11	**0.001**
		AP-VMAT	**6 ± 2**	3–9	
	D_0.03 cc_ [Gy]	HT	41 ± 7	25–53	0.17
		AP-VMAT	36 ± 10	18–52	
Spinal Cord	D_mean_ [Gy]	HT	4.1 ± 1.6	1.6–7.9	**< 0.001**
		AP-VMAT	**2.0 ± 0.5**	1.0-2.7	
	D_0.03 cc_ [Gy]	HT	24 ± 7	10–34	0.6
		AP-VMAT	10 ± 4	3–21	

V_XGy_ = percentage of volume receiving XGy; V_X%_ = percentage of volume receiving X% of the prescription isodose; D_X%_ = dose to X% of the volume; D_Xcc_ = dose to Xcc of the volume; D_mean_ = mean dose; SD = standard deviation. P-values are in bold when indicating a statistically significant difference. When values are statistically significant, the one expressing an improvement is evidenced in bold.

## Discussion

The aim of this study was to compare manually generated HT plans with VMAT plans automatically produced using mCycle for 25 locally advanced breast cancer patients. There are very few published applications of mCycle, such as on prostate cancer, prostate stereotactic body RT, bilateral head-and-neck cancer, and rectal cancer treated at an MR-Linac [22], head-and-neck cancer [23], and cervical cancer [24]. To the best of our knowledge, no published experience regarding VMAT breast cancer treatment with this system has been reported thus far.

The complexity of breast cancer treatment, which involves shaping the target and ensuring minimal doses to OARs such as the heart, makes the automation of planning processes crucial for workload reduction and treatment quality improvement. Our study demonstrates the feasibility of defining an mCycle wish-list capable of generating VMAT plans that are at least comparable to HT plans. As expected, the HT plans exhibited smaller values for PTV V_105%_, particularly for PTV breast/CW and PTV III-IV. HT dose distributions generally showed higher homogeneity compared to VMAT [25]. No statistically significant differences were observed in PTV V_95%_, except for PTV IMN, where achieving adequate coverage is particularly challenging due to the proximity to the heart.

While HT plans have consistently demonstrated high PTV homogeneity, the wish-list translated into the mCycle planning process explicitly prioritized homogeneity for AP-VMAT plans for all PTVs, including the IMN, as a Priority 1 objective. Control over point hotspots, which were subject to the selected Monte Carlo variance (1% per plan), was also emphasized to strike a balance between accuracy and optimization speed.

No statistically significant differences were observed for ipsilateral organs, while AP-VMAT showed advantages over HT in terms of OARs D_mean_ (p < 0.05) for all other organs. While the nature of HT delivery may explain this finding for the contralateral lung and contralateral breast [26], the manual planner likely did not further optimize the spinal cord and esophagus, stopping just below the constraints. This represents one of the main advantages of AP over manual planning: the ability to consider all possible OARs and achieve additional gains at no extra cost. This capability of further reducing the dose to an OAR, even below the constraints or objective goals, is particularly valuable in the context of patient retreatment.

Significantly, the developed wish-list demonstrated applicability across the entire heterogeneous patient sample in terms of target volumes and doses. The study also highlighted the ease of adapting the wish-list to protocol changes, such as adding a volume (e.g., IMN) or altering prescription doses by introducing a SIB. One of the limitations of the study is that there is only one breast patient per side in the validation set. Therefore, the results for this subset of patients cannot be considered definitive and should be approached with caution.

Another limitation of our study is the comparison of plans calculated using different dose algorithms (Monte Carlo for research Monaco and Collapsed Cone Convolution for HT). However, the primary focus of this work was not to directly compare the two planning modalities but to evaluate the clinical feasibility of the AP-VMAT method as an alternative to HT for breast treatment with lymph nodes. It is worth noting that the QA analysis surpassed the usual clinical acceptability for all analyzed cases.

Our study findings align with the results of Biston et al. [23], which demonstrated the overall superiority of mCycle compared to HT plans for head and neck cancer. They also reported that no manual adjustments to the wish-lists were necessary to achieve robust clinical plans, despite the cohorts being highly heterogeneous in terms of anatomy, volume, and tumor location.

## Conclusions

The implementation of the mCycle AP software demonstrated its capability to automate the planning process for VMAT treatment of breast/CW and nodal irradiation, significantly reducing the planning workload. Comparing the AP-VMAT plans with the clinical HT plans revealed comparable or improved quality. The developed wish-list proved to be robust, accommodating minor variations in RT protocols and anatomical differences between patients. The auto-VMAT plans exhibited complete deliverability and consistent dosimetry.

Furthermore, it is important to document the experiences of Monaco users (apart from the software developers) in creating the wish-list that drives the mCycle optimization process.

### Electronic supplementary material

Below is the link to the electronic supplementary material.

Supplementary Material 1

Supplementary Material 2

Supplementary Material 3

Supplementary Material 4

## Data Availability

The data used in this study can be made available for scientific purposes previous approval of all Authors

## Abbreviations

VMAT: Volumetric Modulated Arc Therapy
HT: Helical Tomotherapy
IMN: Internal Mammary Node
SIB: Simultaneous Integrated Boost
OAR: Organs At Risk
PTV: Planning Target Volume
AP: Automatic Planning
MCO: Multi Criteria Optimization
RT: Radiation Therapy
TPS: Treatment Planning System
CT: Computed Tomography
CTV: Clinical Target Volume
