# Supplementary material for: Fully automated volumetric modulated arc therapy technique for radiation therapy of locally advanced breast cancer
Source: Radiat Oncol. 2023 Oct 30;18:176. doi: 10.1186/s13014-023-02364-8 (PMC10617151; doi:10.1186/s13014-023-02364-8)
Supplement: Supplementary file 1 — Supplementary Material 1 [file 13014_2023_2364_MOESM1_ESM.docx]

**Table S1**. Main clinical features of the series.

| **Feature** | **N (%)** |
| --- | --- |
| *Breast Surgery*  Breast-conserving surgery  Mastectomy | 2 (8)  23 (92) |
| *Axillary surgery*  SLNB  ALND | 2 (8)  23 (92) |
| *Postoperative T stage*  pTx/pTis  pT1-2  pT3-4 | 7 (28)  14 (56)  4 (16) |
| *Postoperative N stage*  pN0  pN1  pN2-3 | 6 (24)  4 (16)  15 (60) |
| *Tumor grade*  G1-2  G3 | 5 (20)  20 (80) |
| *Hormonal receptors status°*  Positive  Negative | 18 (72)  7 (28) |
| *HER2 status*  Negative  Positive | 18 (72)  7 (28) |
| *Lymph vascular invasion*  Absence  Presence | 15 (60)  10 (40) |
| *DCIS component*  Absence  Presence | 21 (84)  4 (16) |
| *Chemotherapy*  Neoadjuvant  Neoadjuvant and adjuvant  Adjuvant  None | 9 (36)  5 (20)  9 (36)  2 (8) |
| *Adjuvant endocrine therapy*  Yes  No | 18 (72)  7 (28) |

Abbreviations = SLNB, sentinel lymph node biopsy; ALND, axillary lymph nodes dissection; G, grade; HER2, Human Epidermal Growth Factor Receptor 2; DCIS, ductal carcinoma in situ.

°Hormonal receptor positive status is defined positive if estrogen receptor (ER) and/or progesterone receptor (PgR) equal or more than 10%.
