# Supplementary material for: Fully automated volumetric modulated arc therapy technique for radiation therapy of locally advanced breast cancer
Source: Radiat Oncol. 2023 Oct 30;18:176. doi: 10.1186/s13014-023-02364-8 (PMC10617151; doi:10.1186/s13014-023-02364-8)
Supplement: Supplementary file 2 — Supplementary Material 2 [file 13014_2023_2364_MOESM2_ESM.docx]

| Structure | Cost Function | Priority | Dose (Gy) | RMS Dose excess (Gy) | Power Law exponent | Mean Organ Damage (%) | Shrink margin (cm) | Sufficient (Gy) |
| --- | --- | --- | --- | --- | --- | --- | --- | --- |
| PTV breast/CW | Target Penalty | 1 | 47.5 (96% PTV) |  |  |  |  | 48 |
|  | Quadratic Overdose | Clinical Constraint | 52.5 | 0.05 |  |  |  |  |
|  | Target Penalty | 4 | 50 (50% PTV) |  |  |  |  | 51 |
| PTV III-IV | Target Penalty | 1 | 47.5 (96% PTV) |  |  |  |  | 48 |
|  | Quadratic Overdose | Clinical Constraint | 52.5 | 0.05 |  |  |  |  |
|  | Target Penalty | 4 | 50 (50% PTV) |  |  |  |  | 51 |
| PTV IMN | Target Penalty | 1 | 47.5 (96% PTV) |  |  |  |  | 48 |
|  | Quadratic Overdose | Clinical Constraint | 52.5 | 0.05 |  |  |  |  |
|  | Target Penalty | 4 | 50 (50% PTV) |  |  |  |  | 51 |
| Patient | Quadratic Overdose | Planning Constraint | 47.5 | 0.02 |  |  | 0.3 |  |
|  | Quadratic Overdose | Planning Constraint | 30 | 1.4 |  |  | 0.5 |  |
|  | Maximum Dose | Planning Constraint | 55 |  |  |  |  |  |
|  | Conformality | 8 | 1 |  |  |  |  |  |
|  | Quadratic Overdose | Planning Constraint | 19 | 0.05 |  |  | 4.0 (PTV breast/CW)  5.0 (PTV III-IV) |  |
| Heart | Serial | Clinical Constraint | 4 |  | 1 |  |  |  |
|  | Serial | 6 | 1 |  | 1 |  |  |  |
|  | Parallel | 6 | 2 |  | 1 | 5 |  |  |
| Contralateral breast | Serial | 7 | 10 |  | 1 |  |  | 6.5 |
|  | Maximum Dose | Clinical constraint | 25.8 |  |  |  |  |  |
| Ipsilateral lung | Serial | 5 | 10 |  | 1 |  |  |  |
|  | Parallel | 3 | 16 |  | 3.5 | 30 |  |  |
|  | Parallel | 3 | 8 |  | 3.5 | 30 |  |  |
|  | Parallel | 3 | 5 |  | 4 | 10 |  |  |
| Contralateral lung | Parallel | 3 | 5 |  | 4 | 10 |  |  |
| Esophagus | Parallel | 6 | 45 |  | 3 | 33 |  |  |
| Spinal cord | Quadratic Overdose | 2 | 10 | 0.02 |  |  |  |  |
|  | Serial | 5 | 2 |  | 1 |  |  |  |

**Table S2.** Wish-list for the single dose plan.

Table S2 reports the details of the Wish List that relates to the Cost Functions implemented in Monaco:

- Target Penalty: physical cost function used to prescribe dose to targets.
- Quadratic Overdose: used to limit high doses in the structure to which it is applied.
- Maximum Dose: physical cost function used with either targets or OARs. It is a hard barrier, kicking in “all at once” whenever voxels cross the maximum dose threshold.
- Conformality: physical cost function used for shaping the high dose volume tightly around one or several target volumes.
- Serial: biological cost function that applies large penalties for hot spots even if they are small in volume.
- Parallel: biological cost function preferred for parallel OARs. It is the biological equivalent of the DVH constraint.

The Priority column relates to the classification for the prescription as Clinical or Planning Constraints or which degree of priority with #1 being the highest possible one (usually assigned to target coverage). In mathematical terms those priority relates with weights of the cost functions during the optimization process with the Clinical Constraints being the highest ones as those prescriptions whose violations would incur into the non-acceptance of the plan; the Planning Constraints are those prescriptions that planners would normally adopt in terms of gradients requirements and the objective priorities those prescriptions that, if satisfied, would then be turned into constraints and not subjected to violation during the optimization process.

RMS (Root Mean Square) Dose excess is an iso-constraint typical of the Quadratic Cost function defined in Monaco TPS as well as the Power Law Exponent pertinent to the Serial and Parallel Cost Functions.

The Mean Organ Damage is the biological equivalent to the fraction of the volume of the structure that can be sacrificed, and it is pertinent to the Parallel Cost Function.

The Shrink Margin is the Margin from the PTV adopted by any single cost function in Monaco.

The Sufficient Column is related to the mCycle process and is meant to quench the MCO approach if during the optimization a sufficient value is reached as further decrease of example of a tolerance value wouldn’t result in any major clinical benefit but allowing more degree of modulation for the optimization of the remaining cost functions.
