# Supplementary material for: Fully automated volumetric modulated arc therapy technique for radiation therapy of locally advanced breast cancer
Source: Radiat Oncol. 2023 Oct 30;18:176. doi: 10.1186/s13014-023-02364-8 (PMC10617151; doi:10.1186/s13014-023-02364-8)
Supplement: Supplementary file 4 — Supplementary Material 4 [file 13014_2023_2364_MOESM4_ESM.docx]

| **Calculation Properties** | | **Fluence Optimization** | |
| --- | --- | --- | --- |
| Grid Spacing [cm] | 0.30 | Beamlet Width [cm] | 0.30 |
| Statistical Uncertainty | 3 % per Control Point | Fluence Smoothing | High |
|  |  | Target Margin | Normal |
|  |  | Avoidance Margin | Normal |
| **Shape Optimization** | | **Sequencing** | |
| Leaf Seq. Algorithm | Hyperion | Optimization | Segment Shapes and Weights |
| Min. Segment Area [cm²] | 2.0 | High Precisions Leaf | VERO |
| Min. Segment Width [cm] | 0.50 | SSO Algorithm | Hyperion |
| Min. MU/Segment | 4 | SSO loops | 20 |
| Max Segments Per Plan | 12 |  |  |

**Table S4.** Plan parameters used in auto-VMAT plans: calculation properties, fluence optimization, shape optimization and sequencing.
